# Precision (repeatability and reproducibility) of papillary and peripapillary vascular density measurements using optical coherence tomography angiography in children

**DOI:** 10.3389/fmed.2023.1037919

**Published:** 2023-03-23

**Authors:** Ruru Chen, Xinyu Liu, Mingyu Yao, Zhilin Zou, Xinyi Chen, Zheng Li, Xin Chen, Mengjuan Su, Hengli Lian, Weiwei Lu, Yizhou Yang, Colm McAlinden, Qinmei Wang, Shihao Chen, Jinhai Huang

**Affiliations:** ^1^School of Ophthalmology and Optometry and Eye Hospital, Wenzhou Medical University, Wenzhou, Zhejiang, China; ^2^Department of Ophthalmology, Eye Institute, Eye and ENT Hospital, Fudan University, Shanghai, China; ^3^NHC Key Laboratory of Myopia (Fudan University), Key Laboratory of Myopia, Chinese Academy of Medical Sciences, Shanghai, China; ^4^Department of Ophthalmology, Singleton Hospital, Swansea Bay University Health Board, Swansea, United Kingdom; ^5^Department of Ophthalmology, Royal Gwent Hospital, Aneurin Bevan University Health Board, Newport, United Kingdom; ^6^Shanghai Research Center of Ophthalmology and Optometry, Shanghai, China

**Keywords:** vascular density, optical coherence tomography angiography, repeatability, reproducibility, children

## Abstract

**Importance:**

Optical coherence tomography angiography (OCTA) has been widely applied into children, however, few studies have assessed the repeatability and reproducibility of papillary and peripapillary VD in healthy children.

**Objective:**

To assess the precision of papillary and peripapillary vascular density (VD) measurements using optical coherence tomography angiography (OCTA) and analyze the effects of the signal strength index (SSI) and axial length (AL) on precision estimates.

**Design, setting, and participants:**

This was a prospective observational study. Seventy-eight children aged 6–16 years underwent 4.5 × 4.5 mm OCTA (RTVue XR Avanti) disc scans: two scans by one examiner (repeatability) and two additional scans by another examiner (reproducibility). Within-subject standard deviation (Sw), test-retest reproducibility (TRT), within-subject coefficient of variation (CoV), intraclass correlation coefficient (ICC), and Bland–Altman analysis were performed.

**Main outcomes and measures:**

In repeatability measurement, the fluctuation ranges (minimum to maximum) of VD between intraexaminer A/B in Sw, TRT, CoV, and ICC were (1.05–2.17)% / (1.16–2.32)%, (2.9–6)% / (3.21–6.44)%, (1.9–4.47)% / (2.08–5)%, and (0.588–0.783)% / (0.633–0.803)%, respectively. In reproducibility measurement, the fluctuation ranges of VD in Sw, TRT, CoV, and ICC were 1.11–2.13%, 3.07–5.91%, 1.99–4.41%, and 0.644–0.777%, respectively. VD was negatively correlated with SSI in most sectors of the peripapillary (e.g., inferior nasal, temporal inferior, temporal superior, superior temporal, and superior nasal). AL was positively correlated with inferior temporal VD and negatively correlated with superior nasal VD.

**Conclusion and relevance:**

Optical coherence tomography angiography showed moderate-to-good repeatability and reproducibility for papillary and peripapillary perfusion measurements in healthy children. The SSI value affects most of the peripapillary VD, while AL affects only the temporal inferior and nasal superior peripapillary VD.

## Highlights

- To assess the precision of papillary and peripapillary vascular density (VD) measurements using optical coherence tomography angiography (OCTA), 78 children aged 6–16 years underwent 4.5 × 4.5 mm OCTA disc scans.
- OCTA showed moderate-to-good repeatability and reproducibility for papillary and peripapillary perfusion measurements in healthy children.

## Introduction

Examination of papillary and peripapillary blood flow in children can help understand the pathological mechanisms of some optic nerve diseases, such as juvenile glaucoma and optic neuritis (1, 2). Consequently, precise estimates of the parameters of the papillary and peripapillary blood flow are imperative.

With the advent of optical coherence tomography angiography (OCTA), it is now possible to non-invasive images and quantify macular, papillary and peripapillary vascular networks through fast scanning and image processing algorithms (3–5). Repeated measurements of blood flow parameters, such as vascular density (VD), will often fluctuate near the true value for the same retinal location of the same individual. It is important to assess the precision of repeated measurements to determine clinical changes in these blood flow parameters; therefore, they are considered when evaluating individual or longitudinal measurements.

Previous studies have shown that OCTA has high repeatability and reproducibility in measuring the area of the foveal avascular zone, superficial and deep retinal VD, and papillary and peripapillary VD in healthy adults (6–11). However, only a few studies have assessed the repeatability and reproducibility of these parameters in healthy children. Zhang et al. (12) reported that OCTA is reliable for evaluating macular perfusion in 8–16 years old children, but the algorithm was not applied to the measurement of papillary and peripapillary VD. Other studies have evaluated the impact of image magnification, associations with ocular biometry, and the effect of patient-specific factors on OCTA measurements (13–15).

In this study, the precision (repeatability and reproducibility) of OCTA was evaluated in measuring papillary and peripapillary VD in 6–16 years old healthy Chinese children in a large sample. Additional objectives were to provide reference values for clinical use and analyze the effects of the signal strength index (SSI) and axial length (AL) on the VD.

## Materials and methods

This prospective observational study was conducted at the Eye Hospital of Wenzhou Medical University. The study protocol was approved by the Medical Ethics Committee of the Eye Hospital of Wenzhou Medical University. Written informed consent was obtained from the legal guardian of each child.

Children aged 6–16 years attending the hospital were invited to participate in this study. The inclusion criteria were as follows: (1) monocular best-corrected distance visual acuity 20/20 or better; (2) refractive spheres between −5.00 D and +5.00 D and cylinder within ± 3 D; (3) intraocular pressure between 10 and 21 mmHg; (4) no retinal pathology; (5) no history of systemic disease.

The papillary and peripapillary VDs were generated by OCTA using the RTVue XR Avanti device (software RTVue, version 2018.1.1.63; Optovue Inc., Fremont, CA, USA) with the Angio Disc (4.5 × 4.5 mm) mode. The measurement principle of angiography and flow imaging has been reported in previous studies (6, 16).

Examiner A (WWL) acquired two repeated scans of the right eye for each patient. Examiner B (LHL) acquired two additional scans of the right eye. Two measurements were taken from previous studies to avoid the fatigue effect in children (17, 18). After the baseline scan was used as the reference scan, the follow-up scan option was chosen to automatically track the same location.

**FIGURE 1:**
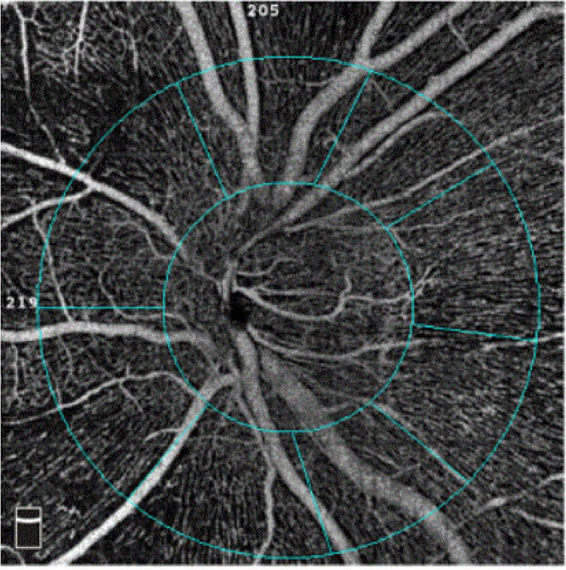
Automatic measured images of optic disc by OCTA with a 4.5 × 4.5 scan pattern.

The software automatically segmented the papillary and peripapillary VD (Figure 1) between the inner limiting membrane and the nerve fiber layer. The VD was defined as the ratio of the area of the large and capillary vessels divided by the total area measured sectors. The capillary VD was the VD, except for the large vessels. According to the Garway-Heath Partition Method, the peripapillary area is subdivided into eight sectors: nasal superior, nasal inferior, inferior nasal, inferior tempo, tempo inferior, tempo superior, superior tempo, and superior nasal. Eyes with poor-quality images with OCTA (quality score < 8), significant motion, or blink artifacts were excluded from the analysis. The AL was measured using an IOLMaster 700 (Zeiss, Germany).

### Statistical analysis

Statistical analyses were performed using Statistical Product and Service Solution (SPSS) statistical software V24.0. Only the right eye was analyzed. Kolmogorov–Smirnov analysis was used to test the normality of the data distribution. Normally distributed variables were described as mean ± standard deviation (SD). Median and interquartile ranges were used to describe non-normally distributed parameters.

The within-subject standard deviation (Sw) (19), test-retest repeatability (TRT) (20, 21), within-subject coefficient of variation (CoV) (22, 23), and intraclass correlation coefficient (ICC) (23, 24) were used to evaluate repeatability and reproducibility. The Sw value was taken as the square root of the residual mean. The TRT value was equal to the Sw multiplied by 2.77. An ICC value > 0.8 or < 0.4 indicates good or poor reproducibility between every two repeated measurements. The CoV value is Sw divided by the average measurement and is expressed as a percentage. A lower CoV indicates greater repeatability (25).

In addition, the Bland–Altman analysis was used to plot the differences in VD and SSI between interexaminer measurements and their means. The method uses the mean difference and 95% limits of agreement (LoAs) between the two examiner measurements. The significance level was set at *P* < 0.05.

To assess interexaminer reproducibility, the two examiners’ mean values between two measurements of each parameter were included in the analysis.

Generalized estimation equations were used to analyze the effects of SSI and AL on precision estimates.

## Results

Eighty-five children were enrolled in this study; seven were excluded due to motion and blink artifacts (two children) or poor image quality (five children). Therefore, the study included 78 children with a median age (interquartile range) of 10 (9–12) years (range, 6–16 years). The mean (± SD) of AL was 24.72 ± 1.26 mm. The mean spherical equivalent refraction was −2.72 ± 1.94 D.

**FIGURE 2:**
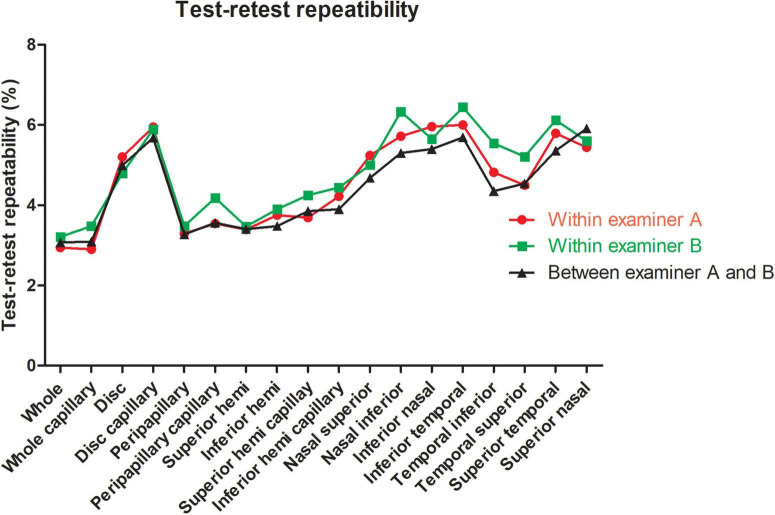
Test-retest repeatability of papillary and peripapillary vascular density measurements intraexaminer and interexaminer.

**FIGURE 3:**
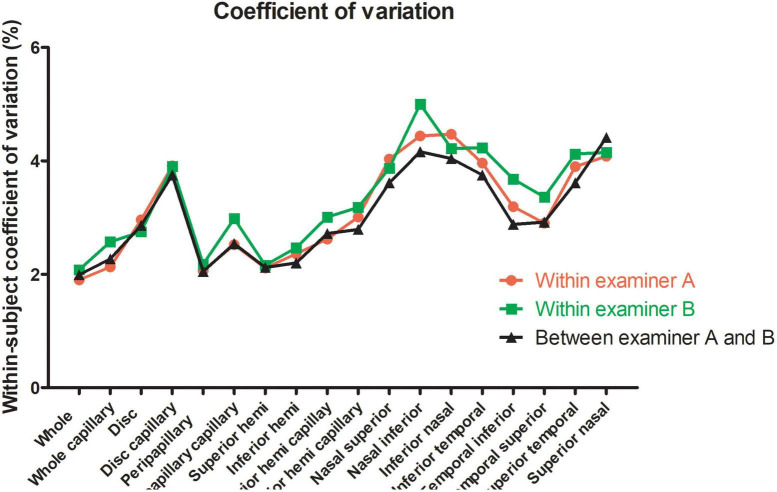
Within-subject coefficient of papillary and peripapillary vascular density measurements intraexaminer and interexaminer.

For the repeatability of two measurements within intra-examiners A and B, the mean ± SD, Sw, TRT, CoV, and ICC of SSI of the whole images were 73.57 ± 8.03 and 72.46 ± 8.02, 3.91, and 3.49, 10.83, and 9.66, 5.31, and 4.81, 0.788 (0.686–0.859), and 0.827 (0.741–0.886), respectively. Table 1 and Figures 2, 3 show the intraexaminer repeatability in measuring papillary and peripapillary VD. The fluctuation ranges of VD between examiners A and B in Sw, TRT, CoV, and ICC were (1.05–2.17)% / (1.16–2.32)%, (2.9–6)% / (3.21–6.44)%, (1.9–4.47)% / (2.08–5)%, and (0.588–0.783) / (0.633–0.803), respectively.

**TABLE 1:** Intra-examiner repeatability of the OCTA in measuring the papillary and peripapillary vascular density.

Parameter	Examiner	Mean ± SD (%)	Sw (%)	TRT (%)	CoV (%)	ICC (95% CI)
Whole	A	56.01 ± 1.82	1.06	2.94	1.9	0.709 (0.579–0.804)
	B	55.70 ± 1.88	1.16	3.21	2.08	0.681 (0.542–0.785)
Whole capillary	A	49.18 ± 1.82	1.05	2.9	2.13	0.714 (0.586–0.808)
	B	48.94 ± 1.93	1.26	3.48	2.57	0.650 (0.501–0.762)
Disc	A	63.33 ± 2.95	1.88	5.2	2.96	0.588 (0.413–0.721)
	B	62.87 ± 3.24	1.73	4.79	2.75	0.665 (0.515–0.775)
Disc capillary	A	54.94 ± 4.00	2.15	5.95	3.91	0.749 (0.633–0.833)
	B	54.47 ± 4.55	2.13	5.89	3.9	0.803 (0.707–0.869)
Peripapillary	A	57.64 ± 2.21	1.19	3.29	2.06	0.739 (0.613–0.829)
	B	57.44 ± 2.18	1.25	3.48	2.18	0.667 (0.515–0.778)
Peripapillary capillary	A	50.72 ± 2.40	1.28	3.54	2.52	0.757 (0.636–0.841)
	B	50.62 ± 2.44	1.51	4.18	2.98	0.691 (0.546–0.795)
-Superior hemi	A	58.02 ± 2.22	1.22	3.39	2.11	0.746 (0.621–0.834)
	B	57.88 ± 2.23	1.25	3.47	2.16	0.737 (0.609–0.828)
-Inferior hemi	A	57.24 ± 2.34	1.35	3.75	2.36	0.713 (0.584–0.807)
	B	56.96 ± 2.30	1.41	3.9	2.47	0.684 (0.545–0.786)
-Superior hemi capillary	A	50.96 ± 2.54	1.33	3.69	2.62	0.746 (0.629–0.831)
	B	51.01 ± 2.62	1.53	4.25	3.01	0.700 (0.566–0.798)
-Inferior hemi capillary	A	50.58 ± 2.62	1.52	4.22	3.01	0.711 (0.580–0.805)
	B	50.39 ± 2.63	1.6	4.44	3.18	0.686 (0.548–0.788)
-Nasal superior	A	46.98 ± 3.41	1.89	5.24	4.03	0.732 (0.610–0.820)
	B	46.57 ± 3.53	1.8	5	3.87	0.768 (0.659–0.846)
-Nasal inferior	A	46.44 ± 3.98	2.06	5.72	4.44	0.763 (0.652–0.842)
	B	45.67 ± 3.84	2.28	6.33	5	0.700 (0.567–0.797)
-Inferior nasal	A	48.17 ± 4.18	2.15	5.96	4.47	0.765 (0.655–0.844)
	B	48.31 ± 4.10	2.04	5.65	4.22	0.779 (0.674–0.853)
-Inferior temporal	A	54.71 ± 3.58	2.17	6	3.96	0.675 (0.531–0.781)
	B	54.97 ± 3.59	2.32	6.44	4.23	0.633 (0.475 to 0.751)
-Temporal inferior	A	54.64 ± 3.03	1.74	4.82	3.19	0.717 (0.590 to 0.810)
	B	54.39 ± 3.31	2	5.54	3.68	0.690 (0.553–0.791)
-Temporal superior	A	56.05 ± 3.29	1.62	4.5	2.9	0.783 (0.680–0.856)
	B	56.01 ± 3.28	1.88	5.21	3.36	0.719 (0.593–0.811)
-Superior temporal	A	53.51 ± 3.59	2.09	5.79	3.9	0.711 (0.580–0.805)
	B	53.55 ± 3.71	2.21	6.12	4.12	0.699 (0.565–0.797)
-Superior nasal	A	48.19 ± 3.58	1.96	5.44	4.08	0.747 (0.623–0.834)
	B	48.70 ± 3.98	2.02	5.6	4.15	0.785 (0.675–0.860)

SD, standard deviation; Sw, within-subject standard deviation; TRT, test-retest repeatability (2.77 Sw); CoV, within-subject coefficient of variation; ICC, intraclass correlation coefficient.

In the interexaminer reproducibility measurement, the mean ± SD, Sw, TRT, CoV, and ICC of SSI were 73.01 ± 8.02, 4.74, 13.14, 6.50, and 0.652 (0.505–0.763). The fluctuation ranges of VD in Sw, TRT, CoV, and ICC were 1.11–2.13%, 3.07–5.91%, 1.99–4.41%, and 0.644–0.777, respectively, as shown in Table 2 and Figures 2, 3.

**TABLE 2:** Inter-examiner agreement of the OCTA in measuring the papillary and peripapillary vascular density.

Parameter	Mean ± SD (%)	Sw (%)	TRT (%)	CoV (%)	ICC
Whole	55.86 ± 1.85	1.11	3.07	1.99	0.644 (0.494–0.757)
Whole capillary	49.06 ± 1.87	1.11	3.09	2.27	0.648 (0.499–0.760)
Disc	63.10 ± 3.10	1.80	4.99	2.86	0.664 (0.520–0.772)
Disc capillary	54.70 ± 4.28	2.05	5.68	3.75	0.771 (0.663–0.848)
Peripapillary	57.54 ± 2.19	1.18	3.27	2.05	0.711 (0.582–0.805)
Peripapillary capillary	50.67 ± 2.41	1.29	3.56	2.54	0.717 (0.589–0.810)
-Superior hemi	57.95 ± 2.21	1.23	3.40	2.12	0.693 (0.557–0.793)
-Inferior hemi	57.10 ± 2.32	1.26	3.48	2.20	0.708 (0.578–0.803)
-Superior hemi capillary	50.98 ± 2.57	1.39	3.85	2.72	0.709 (0.571–0.808)
-Inferior hemi capillary	50.49 ± 2.62	1.41	3.90	2.79	0.712 (0.583–0.806)
-Nasal superior	46.78 ± 3.46	1.69	4.68	3.61	0.763 (0.653–0.842)
-Nasal inferior	46.05 ± 3.92	1.91	5.30	4.16	0.764 (0.646–0.846)
-Inferior nasal	48.24 ± 4.12	1.95	5.40	4.04	0.777 (0.671–0.852)
-Inferior temporal	54.84 ± 3.58	2.06	5.69	3.75	0.671 (0.529–0.777)
-Temporal inferior	54.51 ± 3.16	1.57	4.35	2.88	0.755 (0.641–0.836)
-Temporal superior	56.03 ± 3.28	1.64	4.54	2.92	0.751 (0.635–0.834)
-Superior temporal	53.53 ± 3.64	1.93	5.36	3.61	0.719 (0.591–0.811)
-Superior nasal	48.45 ± 3.78	2.13	5.91	4.41	0.683 (0.545–0.785)

SD, standard deviation; Sw, within-subject standard deviation; TRT, test-retest reproducibility (2.77 Sw); COV, within-subject coefficient of variation; ICC, intraclass correlation coefficient.

**FIGURE 4:**
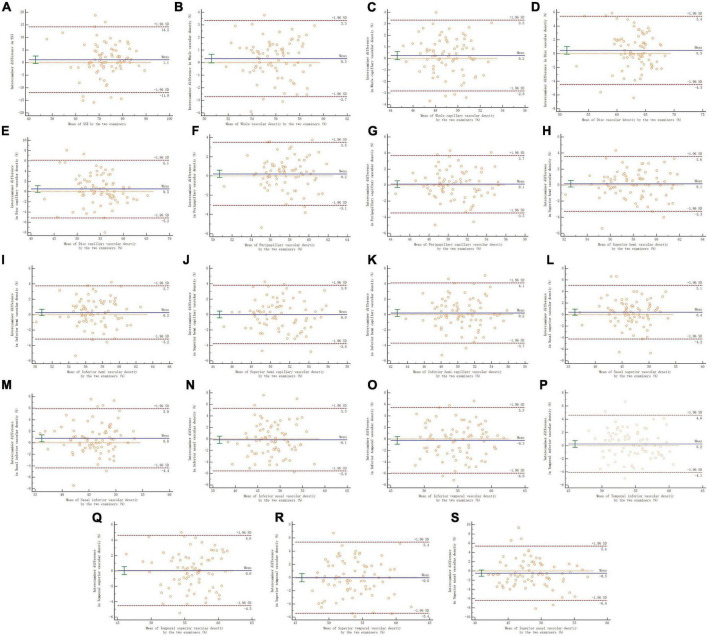
Panels **(A–S)** shows the Bland–Altman plots of reproducibility of interexaminer on papillary and peripapillary vascular density measurement. A solid line represents the mean difference. The upper and lower lines represent the 95% limit of agreement.

In the reproducibility measurement of papillary and peripapillary VD, the interexaminer 95% LoA range for whole, whole capillary, disc, disc capillary, peripapillary, and peripapillary capillary was 6.0, 6.1, 9.9, 11.3, 6.6, and 7.2%, respectively, and other sectors were shown in Figure 4.

The effects of SSI and AL on the repeatability measurements for two examiners of papillary and peripapillary VD are shown in Tables 3, 4. Overall, the disc, disc capillary, and VD increased with increasing SSI. This relationship was the opposite in most sectors of the peripapillary, including the inferior nasal, temporal inferior, temporal superior, superior temporal, and superior nasal. In other sectors, there is no relationship between the two.

**TABLE 3:** Effect of signal strength index on the repeatability measurement of the papillary and peripapillary vascular density.

Vascular density	Coefficient (SE)	95% CI	*P-*value
Whole	0.053 (0.013)	0.027–0.079	<0.001
Whole capillary	0.010 (0.014)	−0.017 to 0.037	0.471
Disc	0.215 (0.019)	0.178–0.252	<0.001
Disc capillary	0.213 (0.028)	0.158–0.267	<0.001
Peripapillary	0.002 (0.016)	−0.029 to 0.034	0.879
Peripapillary capillary	−0.062 (0.017)	−0.096 to −0.028	<0.001
-Superior hemi	0.003 (0.161)	−0.031 to 0.032	0.985
-Inferior hemi	0.004 (0.017)	−0.029 to 0.037	0.812
-Superior hemi capillary	−0.071 (0.018)	−0.106 to −0.036	<0.001
-Inferior hemi capillary	−0.052 (0.019)	−0.090 to −0.015	0.006
-Nasal superior	0.036 (0.025)	−0.013 to 0.085	0.149
-Nasal inferior	0.005 (0.028)	−0.050 to 0.061	0.857
-Inferior nasal	−0.118 (0.029)	−0.175 to −0.061	<0.001
-Inferior temporal	−0.026 (0.026)	−0.077 to 0.026	0.333
-Temporal inferior	−0.075 (0.023)	−0.119 to −0.030	0.001
-Temporal superior	−0.118 (0.023)	−0.162 to −0.073	<0.001
-Superior temporal	−0.096 (0.026)	−0.147 to −0.045	<0.001
-Superior nasal	−0.156 (0.026)	−0.207 to −0.105	<0.001

**TABLE 4:** Effect of axial length on the repeatability measurement of the papillary and peripapillary vascular density.

Vascular density	Coefficient (SE)	95% CI	*P-*value
Whole	−0.121 (0.091)	−0.301 to 0.058	0.184
Whole capillary	−0.007 (0.093)	−0.191 to 0.176	0.936
Disc	−0.052 (0.153)	−0.353 to 0.250	0.735
Disc capillary	0.319 (0.205)	−0.085 to 0.723	0.121
Peripapillary	−0.088 (0.108)	−0.300 to 0.124	0.413
Peripapillary capillary	0.042 (0.119)	−0.193 to −0.277	0.727
-Superior hemi	−0.133 (0.108)	−0.346 to 0.080	0.220
-Inferior hemi	−0.045 (0.115)	−0.272 to 0.182	0.698
-Superior hemi capillary	0.021 (0.124)	−0.223 to 0.265	0.867
-Inferior hemi capillary	0.060 (0.131)	−0.197 to 0.317	0.647
-Nasal superior	−0.340 (0.167)	−0.668 to −0.012	0.042
-Nasal inferior	−0.037 (0.192)	−0.416 to 0.341	0.846
-Inferior nasal	−0.160 (0.202)	−0.557 to 0.237	0.429
-Inferior temporal	0.133 (0.180)	−0.220 to 0.488	0.458
-Temporal inferior	0.399 (0.154)	0.095 to 0.702	0.010
-Temporal superior	0.087 (0.161)	−0.230 to −0.403	0.590
-Superior temporal	0.203 (0.181)	−0.153 to 0.560	0.263
-Superior nasal	0.351 (0.185)	−0.013 to 0.714	0.058

Axial length was positively correlated with VD in the temporal inferior sector, while in the nasal superior sector it was the opposite. In most other sectors, the AL did not correlate with the VD. There was no correlation between age and the repeatability of the papillary and peripapillary vascular density measurement (all *P* > 0.05), except for superior temporal sector in measurement of the examiner A, shown in Table 5.

**TABLE 5:** Effect of age on the repeatability measurement of the papillary and peripapillary vascular density.

	Pearson’s*r*		
**Vascular density**	**Examiners A**	**Examiners B**	**Mean**
Whole	−0.164	0.122	−0.104
Whole capillary	−0.16	0.069	−0.101
Disc	−0.117	−0.062	−0.158
Disc capillary	−0.042	−0.098	−0.078
Peripapillary	−0.146	0.06	0.075
Peripapillary capillary	−0.115	−0.003	0.053
-Superior hemi	−0.11	0.036	0.121
-Inferior hemi	−0.163	0.104	0.061
-Superior hemi capillary	−0.116	−0.003	0.15
-Inferior hemi capillary	−0.126	0.034	0.032
-Nasal superior	−0.061	0.183	0.056
-Nasal inferior	−0.038	0.164	0.172
-Inferior nasal	−0.166	0.05	0.018
-Inferior temporal	−0.147	0.186	0.118
-Temporal inferior	−0.175	0.182	0.011
-Temporal superior	0.126	0.043	−0.016
-Superior temporal	−0.277*	−0.11	0.158
-Superior nasal	−0.132	−0.049	−0.091

*P < 0.05.

## Discussion

Many previous studies have reported that OCTA showed moderate-to-good repeatability and reproducibility in adult measurements with small samples (1, 26–29). Although the OCTA measurement scan is fast, it should also be performed in some patients with poor cooperation, such as children. It is rarely reported in larger samples, particularly in the papillary and peripapillary regions of children. The present study showed moderate-to-good repeatability and reproducibility of the OCTA measurements of papillary and peripapillary VD in healthy Chinese children using comprehensive and systematic evaluation indicators.

In repeatability measurement, Venugopal et al. (26) reported that in 30 normal adult eyes (aged 57 years old), Sw, TRT, CoV, ICC of the papillary and each peripapillary sector were 1.3–2.6%, 3.3–7.0%, 2.4–4.4%, and 0.71–0.85, respectively, for three repeated measurements (1). Manalastas et al. (9) found that in two continuous optic nerve head scans of 15 healthy adults aged approximately 70 years old, the Sw, CoV, and ICC of the papillary and each peripapillary sector were 1.58–3.61%, 2.9–5.8%, and 0.42–0.80, respectively. Consistent with this study, Sw, TRT, CoV, and ICC of the papillary and each peripapillary sector in intraexaminer A/B for two repeated measurements were 1.09–2.17% / 1.16–2.32%, 2.94–6.0% / 3.21–6.44%, 1.9–4.47% / 2.08–5.00%, and 0.588–0.783 / 0.633–0.785. Venugopal et al. (26) reported that a TRT < 6% had no clinical significance. The TRT measured for reproducibility and repeatability in this study were lower than this value. Using the same OCTA equipment, the reliability of repeated measurements of papillary and peripapillary VD in children is close to that in adults.

Capillary density (CD) has a potential value in the evaluation of glaucoma (27). Mansoori et al. (28) visualized the radial peripapillary capillary network using OCTA. Repeatability measurements of CD have also been reported in the adult macula (18, 29, 30). Pappelis and Jansonius (17) reported two measurements of pericapillary CD in 30 healthy adult eyes using Canon OCT-HS 100 angiography; the ICC and TRT were 0.79 and 1.8%, respectively. Consistent with this study, the intra-examiner ICC of examiners A/B was 0.757 / 0.691, and the TRT was 3.54% / 4.18%. This shows that as the operational proficiency of the examiner and cooperation of children increases, the repeatability measurement of pericapillary CD in children approaches that of adults.

Rao et al. (31) found that the use of Cirrus HD-OCT (software version 11.0.0.29946) follow-up scans can make repeatability measurements more reliable compared to non-referenced scans in 33 normal adults (48 eyes), and they reported that the nasal, superior, temporal, and inferior peripapillary were 2.2, 2.2, 2.7, and 1.9% for TRT, 1.8, 2.0, 2.1, and 1.8% for CoV, 0.92, 0.92, 0.85, and 0.97, respectively. Venugopal et al. (26) included 46 eyes of 33 healthy adults using non-referenced high-density scans of RTVue-XR SD-OCT (AngioVue, V.2016.2.0.35) and reported that the repeatability estimates of the papillary and peripapillary sections ranged from 1.1 to 1.8% for Sw, 3.0 to 4.9% for TRT, and 2.0 to 3.1% for CoV. Based on follow-up scanning, this study found that the nasal superior, nasal inferior, inferior nasal, inferior temporal, temporal inferior, temporal superior, superior temporal, and superior nasal peripapillaries of intraexaminer A were 1.89, 2.06, 2.15, 2.17, 1.74, 1.62, 2.09, and 1.96% in Sw; 5.24, 5.72, 5.96, 6, 4.82, 4.5, 5.79, and 5.44% in TRT; 4.03, 4.44, 4.47, 3.96, 3.19, 2.9, 3.9, and 4.08% in CoV; and 0.732, 0.763, 0.765, 0.675, 0.717, 0.783, 0.711, and 0.747 in ICC. Differences in the results may be attributed to different sample sizes, including subjects, scanning mode, devices, and algorithms.

In assessing the reproducibility of interexaminer measurements, She et al. (32) reported that CoV was < 2% and ICC was > 0.8 in different sectors of the peripapillary in normal adults. The Bland–Altman plots showed that the 95% LoA range was 12.2% in the superotemporal, 10.1% in the temporal, 11.3% in the inferotemporal, 12.0% in the inferonasal, 8.0% in the nasal, and 7.4% in the superior peripapillary (32). In our study, CoV and TRT were < 4.5% and < 6%, respectively, and ICC was > 0.64. The Bland–Altman plots showed that the 95% LoA range was 9.3% in the nasal superior, 10.3% in the nasal inferior, 10.9% in the inferior nasal, 11.5% in the inferior temporal, 8.7% in the temporal superior, 9.1% in the temporal superior, and 10.8% in the superior temporal, 11.8% in the superior nasal regions. This shows that the reproducibility measurement for children is acceptable but remains lower than that for adults. The unstable fixation in children may affect the image quality and reliability of the measurement. She et al. (32) also found that the reproducibility and repeatability of measurements in different sectors were different, and this study also had similar findings. However, the reason for this difference is unknown.

Some studies have reported that SSI is positively correlated with macular and peripapillary VD, and clinicians should consider the effect of SSI on VD during follow-up studies (12, 15). Based on repeated measurements, Venugopal et al. (26) claimed that SSI was positively correlated with papillary and sectoral peripapillary VD. Lim et al. (33) showed that SSI was positively correlated with peripapillary microvascular VD through a 3 × 3 mm angiography scan. Interestingly, in the sample of children in this study, considering the two examiners repeated measurement factors, SSI was negatively correlated with VD in most peripapillary sectors. Similarly, Rao et al. (31) used Cirrus OCTA and found that SSI was only negatively correlated with peripapillary temporal VD, while there was no correlation in other sectors. Due to differences in scan area, partition algorithm, equipment, and included subjects, further research is needed on the influence of SSI on peripapillary VD measurement. In addition, the influence of physiological factors, such as small fluctuations in perfusion pressure, should also be considered.

Based on repeatability measurements, Lei et al. (34) reported that AL was only positively correlated with peripapillary temporal VD in Optovue and Triton OCTA; this correlation did not exist in other sectors. In the Spectralis and Cirrus OCTA, the positive correlation between AL and VD was reflected in the superior and inferior sectors, and there was no such correlation in other sectors (34). This study also showed that AL was only positively correlated with inferior temporal VD in Optovue OCTA. However, previous studies have demonstrated a negative correlation between peripapillary VD and AL (35, 36), and Sampson et al. (13) suggested that attention should be paid to the correction of AL in VD calculations. This study included healthy children and excluded subjects with high myopia and analyzed them based on repeated measurements by two examiners, which may be the source of differences in the results of the study.

This study has some limitations. First, this study only included Chinese children who used an OCTA device. The reliability of children’s measurements of multiple devices, including other ethnic groups, requires further research. Second, this study removed images with low-quality scores or significant motion and blinking artifacts. Previous studies have reported a large number of low quality OCTA images (37, 38). The latest version of the OCTA algorithm reduces image defects using real-time tracking. Finally, the repeatability and reproducibility of OCTA depend on multiple factors, including system parameters, imaging protocols, and subject compliance. System parameters include optical or mechanical stability and algorithm robustness. Imaging protocols include scan location, scan speed, and field of view: wide-field (39) or narrow field. Lastly, young/elderly subjects (26) and ocular disease (40) can lower the OCTA precision. We need to further observe the consistency of the measurements with a long-term follow-up.

This study reports the reliability of papillary and peripapillary VD measurements in healthy Chinese children. Our results demonstrated low intraexaminer and interexaminer variations in papillary and peripapillary perfusion parameters. Considering the factors of repeatability measurement by the two examiners, SSI affects most of the peripapillary VD, while AL only affects the temporal inferior and nasal peripapillary VD.

## Data availability statement

The original contributions presented in this study are included in the article/supplementary material, further inquiries can be directed to the corresponding authors.

## Ethics statement

The studies involving human participants were reviewed and approved by the Eye Hospital of Wenzhou Medical University. Written informed consent to participate in this study was provided by the participants’ legal guardian/next of kin. Written informed consent was obtained from the individual(s), and minor(s)’ legal guardian/next of kin, for the publication of any potentially identifiable images or data included in this article.

## Author contributions

RC, XL, MY, QW, SC, and JH contributed to the conception and design of the study. ZZ, XyC, ZL, XC, and MS organized the database. HL performed the statistical analysis. RC wrote the first draft of the manuscript. WL, YY, CM, QW, SC, and JH wrote sections of the manuscript. All authors contributed to manuscript revision, read, and approved the submitted version.
